# Ultrasound morphological patterns of testicular tumours, correlation with histopathology

**DOI:** 10.1002/jmrs.426

**Published:** 2020-09-01

**Authors:** Martin Necas, Muthappan Muthupalaniappaan, Cecilia Barnard

**Affiliations:** ^1^ Department of Ultrasound, Radiology Waikato Hospital Hamilton New Zealand

**Keywords:** Scrotum, testis, tumour, ultrasound

## Abstract

**Introduction:**

Ultrasound (US) plays a key role in the detection of testicular tumours. However, reliable characterisation of testicular tumours with US is difficult. The purpose of this study was to investigate the morphological patterns of testicular tumours as seen on modern US imaging and correlate these with histology.

**Methods:**

The imaging features of 50 testicular tumours were analysed and compared with histology. The US appearance was categorized into 15 distinct morphological patterns.

**Results:**

Patient’s age ranged from 0.5 to 85 years. Of the 50 tumours in our series, 49 were malignant. Nearly half of the malignancies were seminomatous germ cell tumours (SGCTs). Tumours ranged in size from 10 to 130 mm with considerable overlap of size between tumours of different histological type. Even small (10 mm) tumours in our cohort were malignant. SGCTs demonstrated a narrower range of morphological appearances than non‐seminomatous germ cell tumours (NSGCTs). Calcification was common in both SGCT and NSGCTs. Multicomponent cystic‐solid appearance was only seen in NSGCTs.

**Conclusion:**

The differentiation of testicular tumours with US continues to be challenging. In this paper, we have demonstrated the diverse morphological patterns of testicular neoplasms and have proposed the study of tumour morphological features as a promising research direction.

## Graphical abstract

Ultrasound (US) plays a key role in the detection of testicular tumours. However, reliable characterisation of testicular tumours with US is difficult. In this paper, we have demonstrated the diverse morphological patterns of testicular neoplasms and have proposed the study of tumour morphological features as a promising novel research direction.

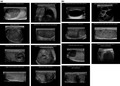

## Introduction

Ultrasound (US) has been the primary imaging modality for the detection of testicular tumours for nearly 4 decades owing to its perfect sensitivity combined with low cost, ease of access and patient acceptance.^1^, ^2^ The differentiation of tumours with US, however, remains challenging due to significant overlap of imaging features between different tumour types.^1^, ^3^, ^4^, ^5^ Because different testicular tumours are associated with substantially different management pathways, prognosis and patient outcomes,^6^ it is desirable that the US examination provides the greatest level of tumour characterisation possible.

In other US applications, morphological features of masses have been successfully incorporated into taxonomies and clinical guidelines in diverse clinical areas such as thyroid nodules (ACR‐TIRADS,^7^ ATA^8^), ovarian tumours (IOTA^9^) and endometrial pathologies (IETA^10^). No uniform morphological taxonomy system to describe testicular tumours currently exists.^3^

Various authors have correlated US features of testicular tumours to histology^11^, ^12^ by analysing a defined set of US features such as size, echogenicity, complexity, margins and presence of calcification^3^, ^11^, ^13^ or matching tumour morphology to pictograms.^12^ For example, there is a general consensus that seminomatous germ cell tumours (SGCT) tend to be hypoechoic and more uniform in architecture than non‐seminomatous germ cell tumours (NSGCT) which often contain solid and cystic components and calcification; however, these observations are not absolute.^14^ With recent advances in US technology, testicular tumours can be visualised in ever increasing detail. While the current guideline for scrotal imaging recommend the use of 7–15 MHz transducers, ultra‐broadband transducers in the 4–18 MHz range are routinely used today.^15^ It is feasible that new and previously unidentified morphological features may be detectable on US. The purpose of this study was to review the US features of 50 testicular tumours, evaluate their US morphological features and correlate the findings with histology.

## Methodology

This was a single‐centre retrospective review of 50 testicular tumours in 2910 patients presenting for US imaging to a tertiary teaching hospital (Waikato Hospital, Hamilton, New Zealand) between the dates of 1/3/2014 and 28/2/2019. Patients were identified using a sequential search of the radiology picture archiving and communication system (Philips PACS Enterprise). The US examinations were performed by hospital‐based qualified sonographers with postgraduate diploma or master’s level qualifications using Philips Epiq 7 or Philips IU22 systems (Philips Healthcare, WA, USA). Patient’s demographics, clinical details, imaging records, surgical letters and histology reports were reviewed. US images and real‐time video clips were reviewed, and the morphological features of the tumours were categorised into distinct morphological patterns. Tumours that exceeded the normal size of the testis were categorised as 'large'. The study was approved by the Health and Disability Ethics Committees, Ministry of Health, New Zealand, reference: 19/CEN/151.

## Results

Fifty testicular tumours were identified in 2910 patients aged between 6 months and 85 years (mean = 39, median = 34). In the patients with testicular tumours, 58 symptoms were provided as an indication for US imaging including swelling in 37 (64%), pain in 12 (21%) and palpable mass in 9(16%) patients. Of the 50 tumours in our series, 49(98%) were malignant and 1 (2%) benign. SGCT represented approximately half of all malignant tumours (Table 1). Nearly all patients (*n* = 45, 98%) who received surgical treatment were treated by radical orchidectomy and only one by partial orchidectomy (2%). One patient with lymphoma received chemotherapy, two patients with metastatic NSGCT died shortly after the diagnosis, and one patient with metastatic thymus cancer received radiotherapy but later died.

**Table 1:** The types of tumours by histological type and patient age

Tumour type	Count, (%)	Patient age in years range (mean, median)
All malignant tumours	49 (98%)	0.5–85 (38, 34)
SGCT	22 (46%)	25–70 (42, 40)
NSGCT	21 (48%)	0.5–59 (28, 28)
Mixed germ cell tumour	15 (30%)	17–38 (27, 27)
Teratoma	3 (6%)	0.5–28 (16, 20)
Choriocarcinoma	2 (4%)	31–34 (33, 33)
Yolk sac tumour	1 (2%)	59
Lymphoma	4 (8%)	41–84 (63, 62)
Metastasis (thymus primary)	1 (2%)	45
Merkel cell tumour	1 (2%)	66
Sertoli (sclerosing)	1 (2%)	57

SGCT, seminomatous germ cell tumours; NSGCT, non‐seminomatous germ cell tumours.

**Figure 1:**
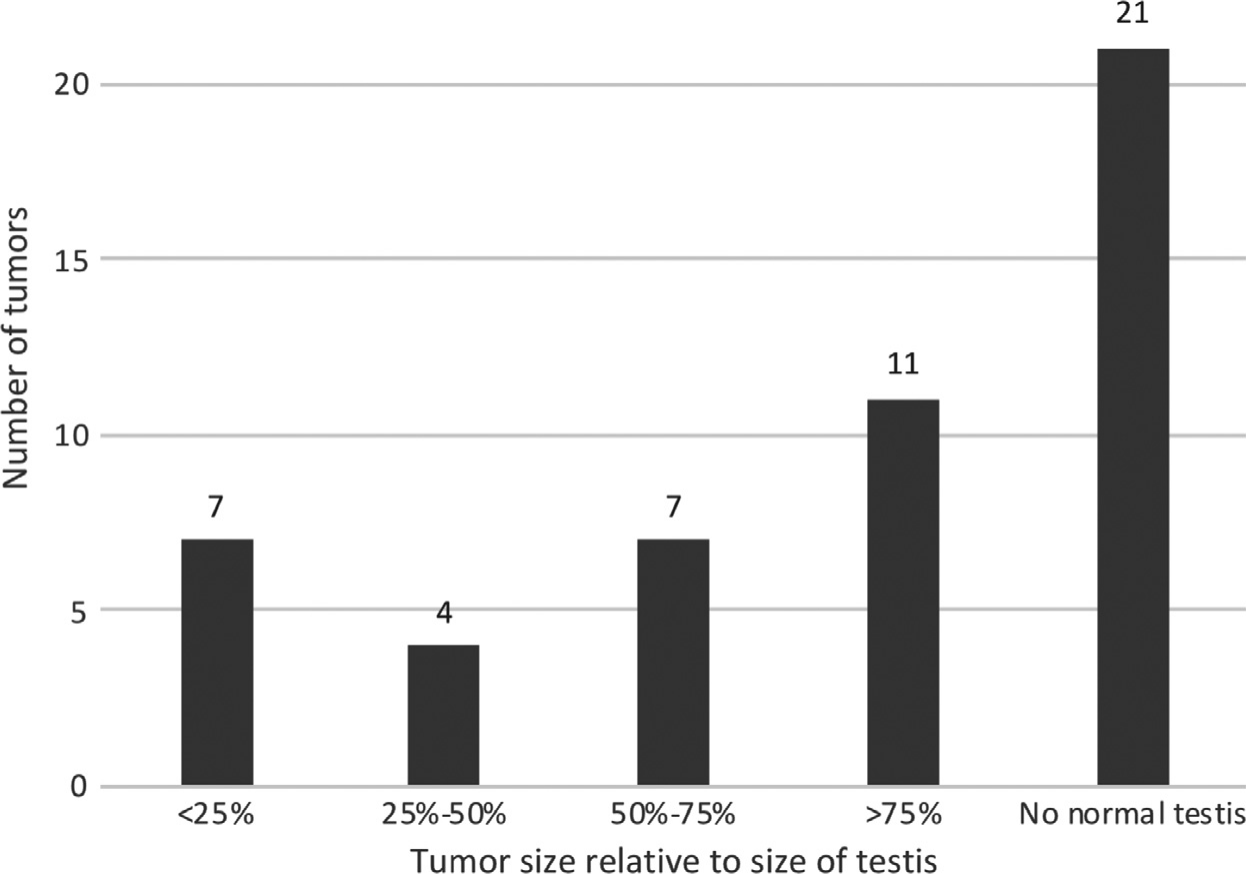
Number of tumours in different size categories based on visual estimation of tumour volume.

**Figure 2:**
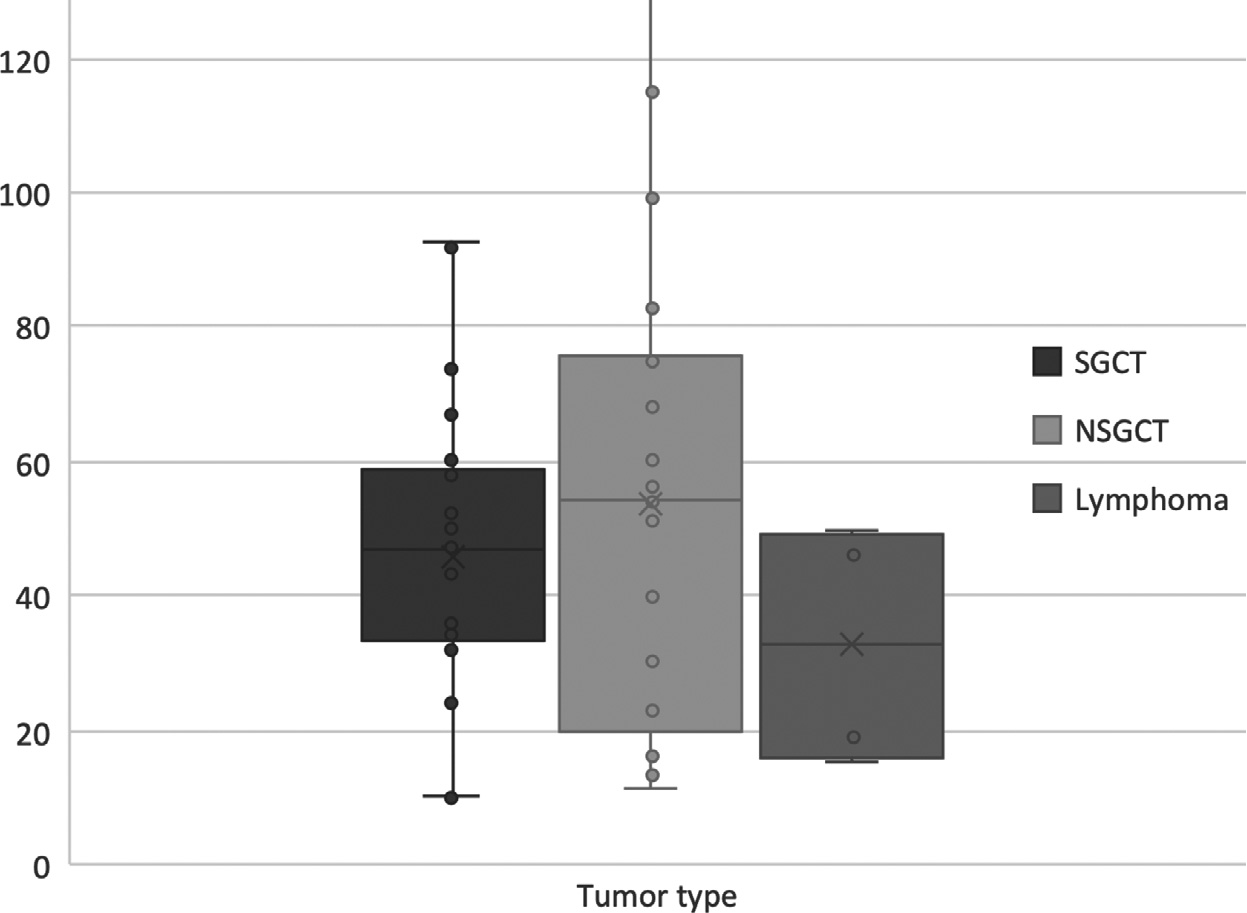
Size distribution of the three most common tumour types.

Tumours in our cohort ranged in size from 10 to 130 mm (mean = 47 mm). By visual estimation, nearly two‐thirds of the tumours involved more than 75% of the testicle (Fig. 1). There was considerable overlap in the size of the tumours (Fig. 2). Both SGCT and NSGCT demonstrated similar distribution of sizes.

**Figure 3:**
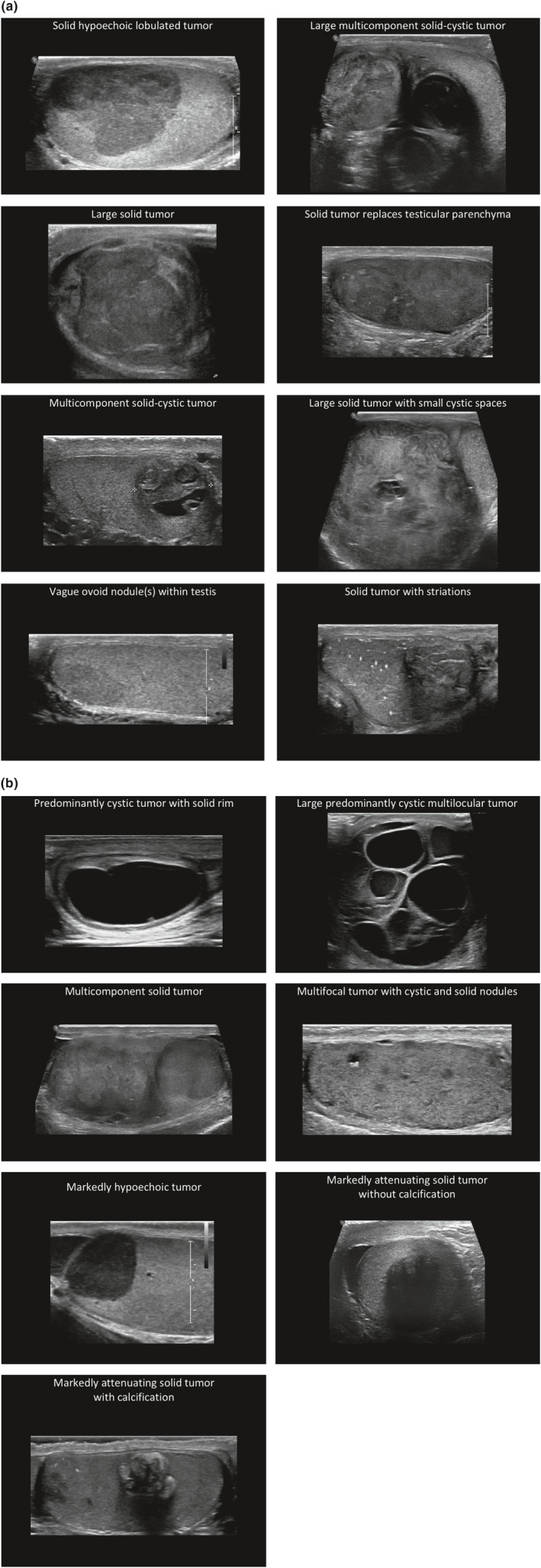
Morphological patterns of testicular tumours as seen on ultrasound.

Tumour morphology was divided into 15 distinct morphological patterns (Fig. 3). The morphological pattern seen on US was related to tumour histology (Table 2). SGCTs demonstrated five morphological patterns, whereas the appearance of NSGCTs was more diverse with 10 morphological patterns. Ten (45%) of the SGCTs showed a classic appearance of a well‐defined hypoechoic relatively homogenous lobulated mass. In no case did a seminoma appear as a multicomponent solid‐cystic lesion. When a multicomponent mass was visualised, it represented a NSGCT in all cases (*n* = 11). SGCTs and NSGCTs appeared as large solid tumours with a similar frequency (23% and 20% respectively).

**Table 2:** The distribution of morphological tumour appearance as seen on ultrasound and histological tumour type

	SGCT (*n*, %)	All NSGCT (*n*, %)	Mixed GCT (*n*, %)	Teratoma (*n*, %)	Choriocarcinoma (*n*, %)	Yolk sac tumour (*n*, %)	Lymphoma (*n*, %)	Sertoli (*n*, %)	Merkel (*n*, %)	Metastasis (*n*, %)
Solid hypoechoic lobulated tumour	10 (45%)	1 (5%)	1 (7%)				1 (25%)			1 (100%)
Large multicomponent solid‐cystic tumour		8 (38%)	7 (47%)			1 (100%)				
Large solid tumour	5 (23%)	3 (14%)	3 (20%)							
Solid tumour replaces testicular parenchyma	3 (14%)								1 (100%)	
Multicomponent solid‐cystic tumour		3 (14%)	2 (13%)		1 (50%)					
Large solid tumour with small cystic spaces	3 (14%)									
Vague ovoid nodules throughout testis	1 (5%)						2 (50%)			
Solid tumour with striations		1 (5%)			1 (50%)					
Predominantly cystic tumour with solid rim		1 (5%)		1(33%)						
Large predominantly cystic multilocular tumour		1 (5%)		1 (33%)						
Multicomponent solid tumour		1 (5%)	1 (7%)							
Multifocal tumour with cystic and solid nodules		1 (5%)	1 (7%)							
Markedly hypoechoic tumour							1 (25%)			
Markedly attenuating solid tumour without calcification								1 (100%)		
Markedly attenuating solid tumour with calcification		1 (5%)		1 (33%)						

GCT, germ cell tumours; NSGCT, non‐seminomatous germ cell tumours; SGCT, seminomatous germ cell tumours.

Calcification was observed in 10 (45%) of SGCT. SGCT with calcification tended to be larger (mean = 52 mm) than SGCT without calcification (mean = 39 mm). Calcification was observed in 14 (67%) of NSGCT. None of the cases of lymphoma, metastasis, Sertoli or Merkle cell tumour demonstrated calcification.

## Discussion

In broad terms, any vascularised mass arising within the testis can be considered a tumour and 98% of testicular tumours are malignant.^16^ Mimics of testicular tumours include segmental infarcts, abscesses, haematomas, regions of fibrosis or granulomas, but these are by their nature avascular.^17^, ^18^ Chronic granulomatous orchitis and fibrous pseudotumour may be confused for a tumour.^14^, ^19^ Adrenal rests may also appear tumour‐like; however, they only occur in the context of congenital adrenal hyperplasia allowing for their differentiation on clinical grounds.^20^ Another intratesticular mimic of tumour is epidermoid cysts which are usually easy to diagnose due to their morphological features.^21^, ^22^ Other rare entities include testicular lipomas, hamartomas and sarcoidosis.^23^

In our cohort, testicular tumour size was quite large which was reflected in the small number of testis‐sparing surgeries. It has been reported that the probability of malignancy decreases with reducing lesion size^24^ with a high percentage of small solid intratesticular lesions being benign.^13^, ^25^, ^26^ We did not encounter many small testicular masses in our patients. All small testicular masses in our cohort were malignant. The only benign tumour was a Sertoli cell tumour measuring 34 mm.

**Figure 4:**
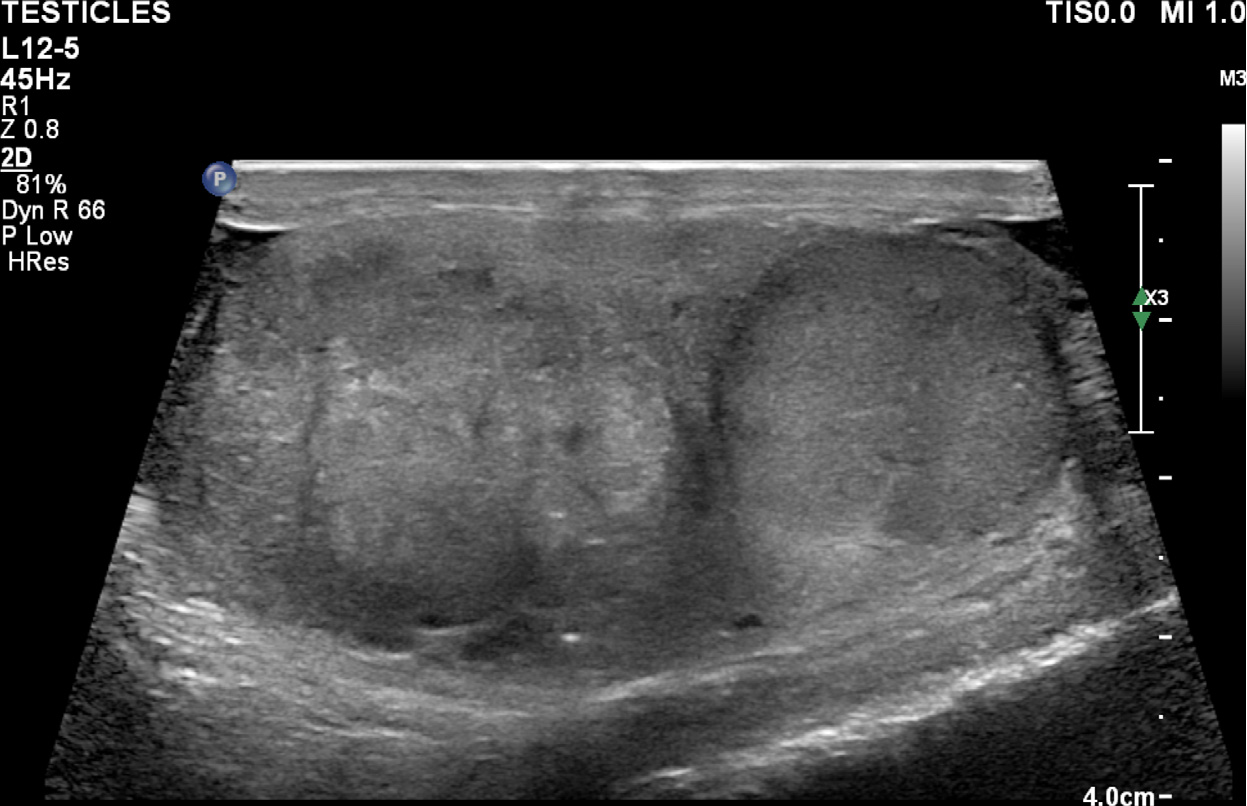
Mixed GCT with a sonographically classic appearance of seminoma in the lower pole and distinctly different tumour component (embryonal cell carcinoma) in the upper pole, histologically confirmed.

Characterisation of testicular morphology with US is difficult. The complexity of testicular tumour appearance is likely multifactorial. First, US provides an acoustic, not histological assessment. The interaction of US with different tissue types is difficult to predict and quantify. Second, increasing tumour size may result in areas of necrosis, haemorrhage and calcification, increasing the morphologic complexity of the tumour. For instance, all small SGCTs in our series were uniformly solid, but large SGCTs appeared more heterogenous and featured cystic spaces and calcifications. Therefore, the appearance of the same tumour type is size‐dependent. Third, testicular NSGCTs and specifically mixed GCTs may contain many different cell lines in various proportions leading to a multicomponent tumour that may or may not contain sonographically distinct elements. In some instances, it is possible to recognise distinct tumour elements within a multicomponent tumour. Figure 4 shows an example of a mixed GCT containing a sonographically classic seminoma component, recognised by the authors and confirmed by histology.

In our analysis of US morphological patterns, we focused on overall tumour morphology rather than individual factors such as echogenicity, heterogeneity, margins and vascularity. This choice was driven by the recognition that most testicular tumours can be said to be heterogenous, margins are very difficult to visualise with confidence, assessment of tunical invasion is not always reliable,^27^ and colour Doppler features cannot be quantified. The use of image samples to aid recognition of pathology has been successfully used in other areas of diagnostic US, specifically in the thyroid.^8^ We therefore propose that the evaluation of US morphological patterns of testicular tumours is a promising novel research field. With high‐resolution US instruments available today, testicular tumours can be studied in ever increasing detail. There is a potential to expand our existing database of tumour morphological patterns by reviewing a larger sample of testicular tumours. This will require a larger dataset, most certainly necessitating a multicentre approach. It may also be possible to employ image analysis tools to add quantitative assessment, for example by examining parameters such as dynamic range, heterogeneity, entropy and other image variables. Furthermore, combining US morphological characteristics with patient’s age, risk factors (previous history of testicular cancer, testicular dysgenesis syndromes such as cryptorchidism, testicular atrophy, mumps orchitis, family history) and tumour markers (AFP, bHCG, LDH) may enable the development of predictive models of tumour type.

Our study has several limitations. Although we reviewed the clinical records of 2910 patients presenting for scrotal US over a 5‐year period, our sample size was only 50 testicular tumours. Because the incidence of some testicular tumours is relatively low, our sample includes only single examples of some tumour types and there are no morphological data on other tumours (Leydig cell) or tumour mimicking entities (fibrous pseudotumour, granuloma). Conversely, our sample contains a Merkle cell tumour of the testis, a tumour so rare, only 8 cases have been published in the literature.^28^ In order to gather a complete set of testicular tumours appearances, a multicentre approach will be required. Second, the description encompasses B‐mode US only and not colour Doppler, contrast‐enhanced US or sonoelastography. This is because B‐mode US is the main modality for tumour detection and assessment. The presence of a tumour always causes architectural distortion of the testis making colour Doppler features difficult to categorise. Colour Doppler by its qualitative nature is difficult to quantify. While some authors^11^ have included arbitrary colour flow scores, colour Doppler does not usually play a role in urological workup at present apart from the binary observation of the presence or absence of vascularity.^3^ Contrast‐enhanced US,^29^ sonoelastography^16^, ^30^ and 3D^31^ are showing some promise for the future, but these techniques are better suited for research purposes and are not readily available in clinical use to the majority of practitioners in Australasia.

## Conclusion

The differentiation of testicular tumours with US continues to be challenging. In this paper, we have demonstrated the diverse morphological patterns of testicular neoplasms and have proposed the study of tumour morphological features as a promising research direction.
